# Turn Back the TIMe: Targeting Tumor Infiltrating Myeloid Cells to Revert Cancer Progression

**DOI:** 10.3389/fimmu.2018.01977

**Published:** 2018-08-31

**Authors:** Robin Maximilian Awad, Yannick De Vlaeminck, Johannes Maebe, Cleo Goyvaerts, Karine Breckpot

**Affiliations:** Laboratory of Molecular and Cellular Therapy, Department of Biomedical Sciences, Vrije Universiteit Brussel, Brussels, Belgium

**Keywords:** cancer, tumor microenvironment, immature myeloid cell, macrophage, dendritic cell, myeloid-derived suppressor cell

## Abstract

Tumor cells frequently produce soluble factors that favor myelopoiesis and recruitment of myeloid cells to the tumor microenvironment (TME). Consequently, the TME of many cancer types is characterized by high infiltration of monocytes, macrophages, dendritic cells and granulocytes. Experimental and clinical studies show that most myeloid cells are kept in an immature state in the TME. These studies further show that tumor-derived factors mold these myeloid cells into cells that support cancer initiation and progression, amongst others by enabling immune evasion, tumor cell survival, proliferation, migration and metastasis. The key role of myeloid cells in cancer is further evidenced by the fact that they negatively impact on virtually all types of cancer therapy. Therefore, tumor-associated myeloid cells have been designated as the culprits in cancer. We review myeloid cells in the TME with a focus on the mechanisms they exploit to support cancer cells. In addition, we provide an overview of approaches that are under investigation to deplete myeloid cells or redirect their function, as these hold promise to overcome resistance to current cancer therapies.

## Introduction to the role of myeloid cells in cancer

Until the beginning of the Twenty-first century, cancer was considered a disease afflicting a single cell. This oversimplified view has made way for a new perspective. As tumors develop, a tireless cross-talk takes place between heterogeneous malignant cells and neighboring parenchymal, stromal, and recruited immune cells. These cells, along with the extracellular matrix, and soluble mediators, form the tumor microenvironment (TME). The composition of the immune infiltrate in the TME largely determines cancer progression and the cancer's sensitivity to various therapies. Tumor-infiltrating CD8^+^ T lymphocytes are key in controlling cancer cells. Consequently, activating *de novo* or existing CD8^+^ T cells using strategies like cancer vaccines and immune checkpoint therapy has attracted considerable attention (1). The durable responses obtained with these therapies have prompted the use of cancer immunotherapy in the standard of care of various cancer types. Despite these vast improvements in cancer treatments, a significant number of patients does not benefit from current cancer immunotherapy. There is ample evidence that myeloid cells, present within the TME, are at the basis of this therapy failure. Therefore, tumor-infiltrating myeloid cells (TIMs) are considered relevant therapeutic targets (2).

Myeloid cells are a heterogeneous group of immune cells that belong to the innate immune system. Among the myeloid cells, monocytes, macrophages, dendritic cells (DCs), and granulocytes have received much attention. These cells, each in their own way, play an essential role in tissue homeostasis. Moreover, monocytes, macrophages and DCs are well known for their ability to regulate T cell responses, thereby bridging innate and adaptive immunity. Tumor cells take advantage of myeloid cells to maintain tissue homeostasis by exploiting the myeloid cells' capacity to produce inflammatory mediators [e.g., interleukin-6 [IL-6] and tumor necrosis factor-α [TNF-α]], growth factors that affect tumor proliferation and vessel formation [e.g., transforming growth factor-β [TGF-β] and vascular endothelial growth factor [VEGF]], and enzymes that degrade matrix proteins [e.g., matrix metallo-proteinases [MMPs]] (3, 4). Moreover, tumor cells take advantage of the myeloid cells' ability to keep T cell responses in check. Thus instructed by tumor cells, myeloid cells aid in creating a TME that is characterized by chronic inflammation, immunosuppression, and continuously proliferating tumor cells that can disseminate. These are important hallmarks of cancer (5). Paradoxically, myeloid cells have also been implicated in the resolution of cancer (4). Myeloid cells can exert profound antitumor functions such as direct tumor cell killing next to indirect tumor cell killing through activation of among others CD8^+^ T cells. In the remainder of the introduction, we discuss the pro- and antitumor properties of different TIM subsets in the context of the cancer immunoediting paradigm (6). We moreover discuss TIMs and how they influence various cancer therapies. Finally we provide an overview of approaches that have been studied to target TIMs and as such enhance the efficacy of current cancer therapies.

## The development and phenotype of tumor-infiltrating myeloid cells in a nutshell

**Figure 1:**
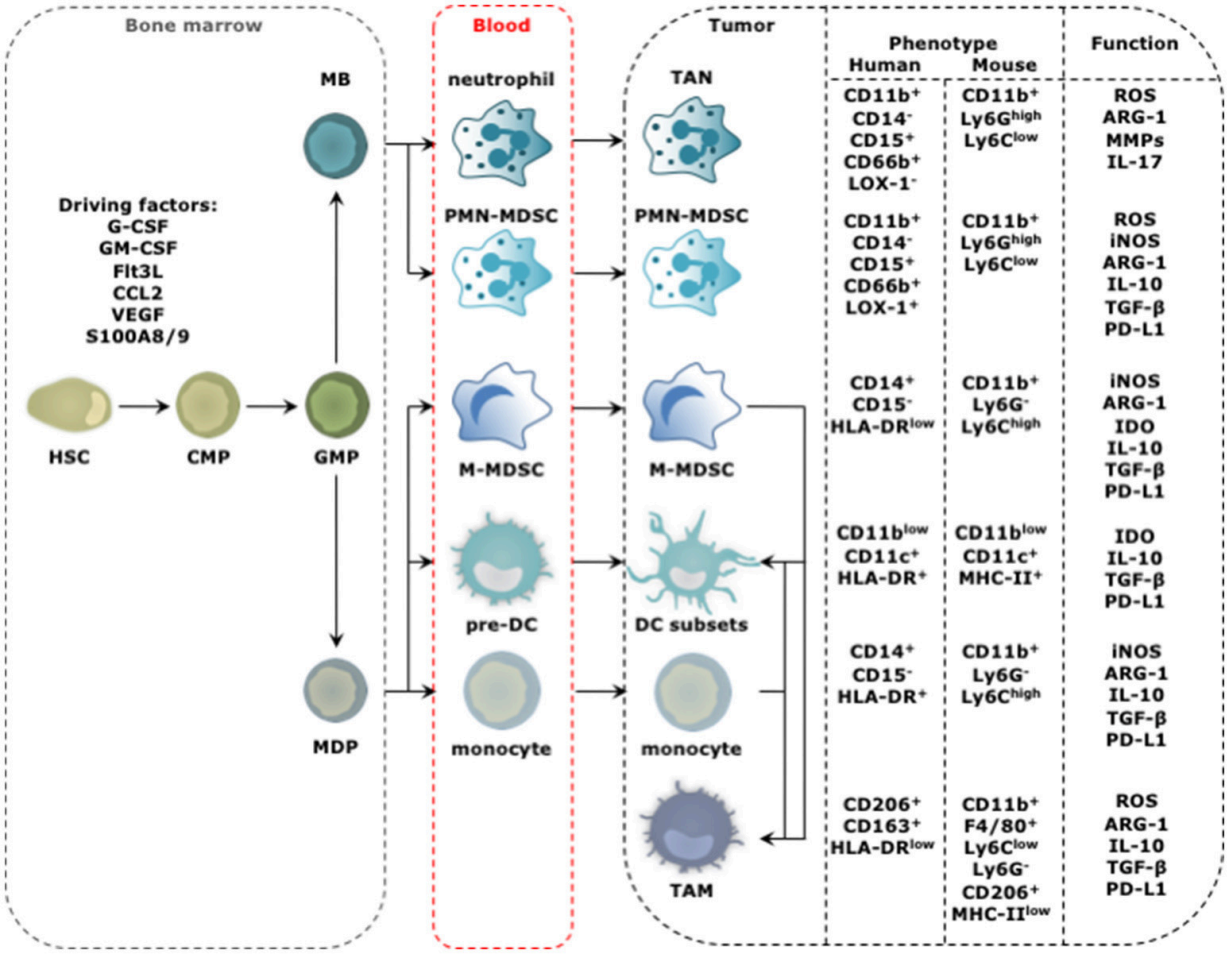
Progression from HSC to tumor-promoting TIM. The distinct steps in the progression from HSC to TIM occur at different locations and start with amplification and differentiation of the HSC and its progenitors, including the common myeloid progenitor (CMP), granulocyte-monocyte progenitor (GMP), myeloblast (MB), and monocyte-dendritic cell progenitor (MDP) in the bone marrow. New myeloid cells are released into the blood stream ready to migrate to the tumor bed. This process is regulated by molecular signals produced by cancer cells and is further amplified by molecular signals produced by among others TIMs. These factors include granulocyte (G) and granulocyte macrophage (GM) colony stimulating factor (CSF), Fms-like tyrosine kinase 3-ligand (Flt3-L), chemokine (C-C motif) ligand 2 (CCL2), VEGF and S100A8/9. The phenotype of TIMs of human and mouse origin, and their functional hallmarks are shown. For TADCs, TANs, and TAMs the figure is simplified as different subsets with either anti- (stimulatory) or protumor (regulatory) functions are discriminated for these cell types. The figure focuses on the subsets with protumor activity.

Tumor-infiltrating myeloid cells (TIMs) constitute a heterogeneous population of cells that are characterized by diversity and plasticity. Many TIMs originate from circulating monocytes and granulocytes, which in turn stem from bone marrow-derived hematopoietic stem cells (HSCs; Figure 1). In the absence of activation signals, persistent stimulation by tumor-derived factors incites monocyte and granulocyte progenitors to divert from their intrinsic pathway of terminal differentiation into mature macrophages, DCs or granulocytes. Instead differentiation into pathological, alternatively activated immature myeloid cells is favored. These immature myeloid cells include tumor-associated DCs (TADCs), tumor-associated neutrophils (TANs), myeloid-derived suppressor cells (MDSCs), and tumor-associated macrophages (TAMs). Alternative to this “emergency myelopoiesis,” TAMs can originate from tissue-resident macrophages, which in turn can be of embryonic or monocytic origin (7–9). These tissue-resident macrophages undergo changes in phenotype and function during carcinogenesis, and proliferation seems key to maintain TAMs derived from tissue-resident macrophages.

Among the TIMs, MDSCs have attracted considerable attention. This TIM population has been divided in polymorphonuclear (PMN) and monocytic (M) MDSCs, which morphologically and phenotypically resemble TANs and monocytes, respectively (10, 11). The most important criterion defining MDSCs is their ability to suppress T cells. Since the tumor is the soil for tumor-promoting myeloid cell types, the term MDSCs and its narrow division into PMN- and M-MDSCs is debatable (3). Two observations argue for a more broad term that gathers immature myeloid cells under the same umbrella. First, several conditions have been described to promote the rapid differentiation of M-MDSCs to either PMN-MDSCs, TAMs or TADCs, highlighting the plasticity of myeloid cells and their ability to shift shape in function of the encountered signals (12–14). Second, the hallmark feature, suppressing T cells, is not unique to MDSCs. Also monocytes, DCs and macrophages can acquire a T cell suppressive phenotype in the TME (3, 15). The above findings hamper pigeonholing of the plastic TIM compartment into discrete subsets but force us to acknowledge the fact that the tumor reigns its environment according to its own rules and molds TIMs that functionally engage in a cross-talk, to serve the tumor. Nonetheless, several TIM subsets can display functions that oppose tumor progression, e.g., activation of tumor-rejecting CD8^+^ T cells. Therefore, we previously proposed to divide TIMs according to their function into myeloid stimulatory cells associated with immune activation, and myeloid regulatory cells associated with immune suppression and wound healing (3).

The functional polarization of TIMs toward aiding or opposing tumor growth is often explained using the T helper (T_H_) 1/2 paradigm of CD4^+^ T cells. In particular TAMs and TANs have been subdivided in so-called type 1 and type 2 TIMs, as such categorizing them into TIMs that exert anti- or protumor activities, respectively (16–21). Although the division in type 1 and type 2 is certainly of interest, it should be emphasized that any form of strict classification does not reflect the complexity of the *in vivo* situation. *In vivo* TIMs are exposed to a multitude of environmental stimuli, which can give opposing cues, resulting in mixed profiles, rather than a strict type 1 or type 2 profile. This has been well documented for TAMs (22–25).

Mature TADCs and type 1 TAMs (TAM1) are considered immunostimulatory TIMs. Both are characterized by high expression of antigen presenting molecules (e.g., MHC-I & -II), co-stimulatory molecules (e.g., CD80 and CD86), next to secretion of high levels of stimulatory cytokines (e.g., IL-6, IL-12, IL-23, and TNF-α) (24). In contrast, MDSCs, type 2 TAMs (TAM2), and tolerogenic TADCs (tolDCs) are considered immunoregulatory TIMs. These share many functional features that enable strong immunosuppression, among which expression of various enzymes like inducible nitric oxide synthase (iNOS), arginase-1 (ARG-1), and indoleamine 2,3 deoxygenase (IDO), secretion of cytokines such as IL-10 and TGF-β, and expression of co-inhibitory molecules like programmed death ligand 1 (PD-L1; Figure 1) (3). Next to immunosuppression, these immunoregulatory TIMs are responsible for tissue remodeling, promotion of tumor cell proliferation and angiogenesis, thereby favoring cancer progression in various ways.

The protumor traits of TIMs are exploited by most tumors through cultivation of regulatory TIMs till large collections of TAM2, MDSCs, TAN2, and small amounts of immature tolDCs are amongst others established. This installs feed forward loops, as many factors produced by regulatory TIMs enforce the generation, recruitment, and function of new regulatory TIMs. These factors include VEGF, TGF-β, S100A8/9 proteins, et cetera. Because the protumor pathways and mechanisms used by regulatory TIMs show redundancy, tackling only one subset or pathway will likely be insufficient to revert cancer progression. In the following sections we discuss the activities of TIMs in more detail in function of the immunoediting paradigm (6).

## Myeloid cells have a cat and mouse relationship with cancer cells

**Figure 2:**
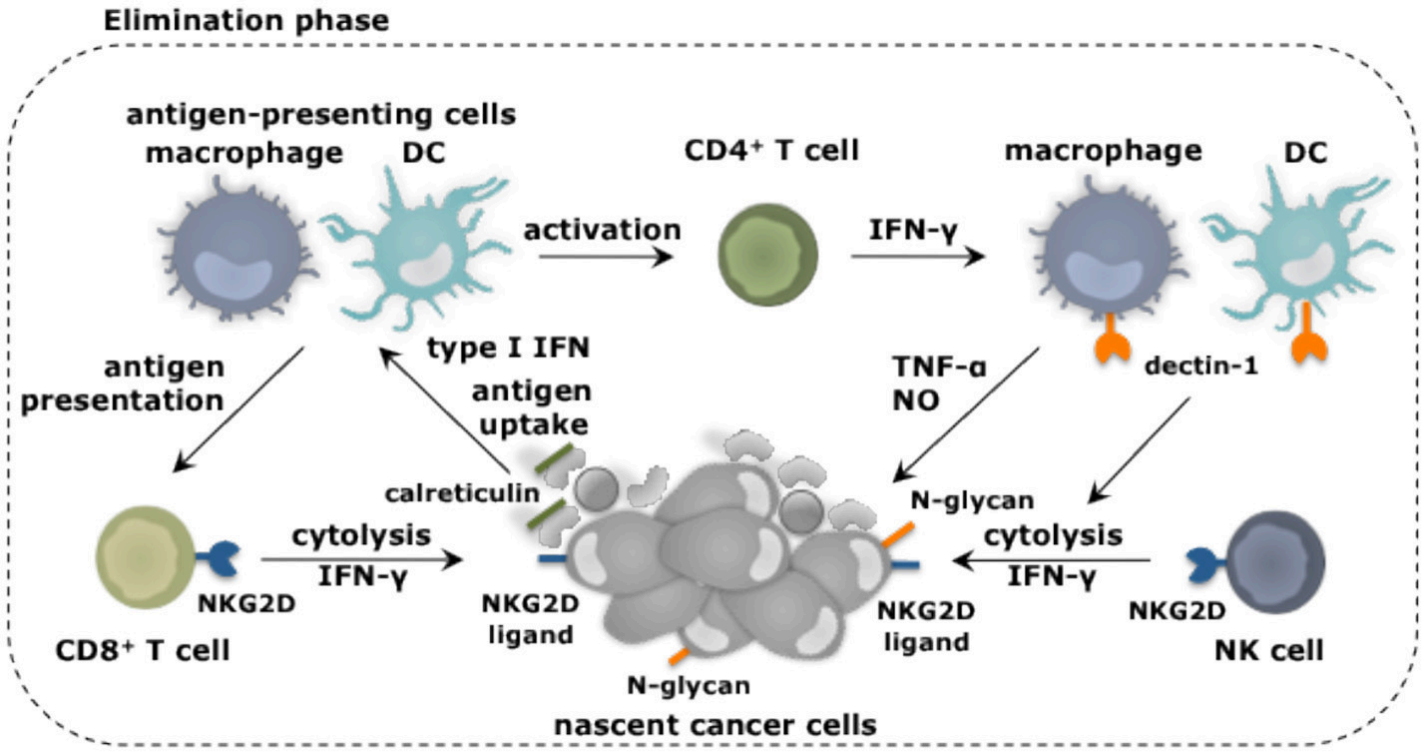
Myeloid cells and their role in elimination of cancer cells. Myeloid cells in addition to other immune cell types fulfill unique as well as redundant functions to achieve tumor cell elimination. Nascent cancer cells are detected by NK cells through the expression of specific ligands, e.g., ligands for the receptor NKG2D. This results in NK cell-mediated killing of the cancer cells, which can be further enhanced through the activation of macrophages and DCs through binding of their receptor dectin-1 to N-glycan structures expressed on certain tumor cells. Cancer cell fragments as well as cancer cells expressing surface calreticulin can be ingested by macrophages (provided that SIRPα is not activated) and DCs. As such these antigen-presenting cells acquire TAAs and can activate CD4^+^ and CD8^+^ T cells. IFN-γ produced by these T cells as well as NK cells is one of the mechanisms exploited to kill cancer cells. This cancer cell killing can be further amplified by activation of the tumoricidal program (NO and TNF-α release) of macrophages via IFN-γ.

**Figure 3:**
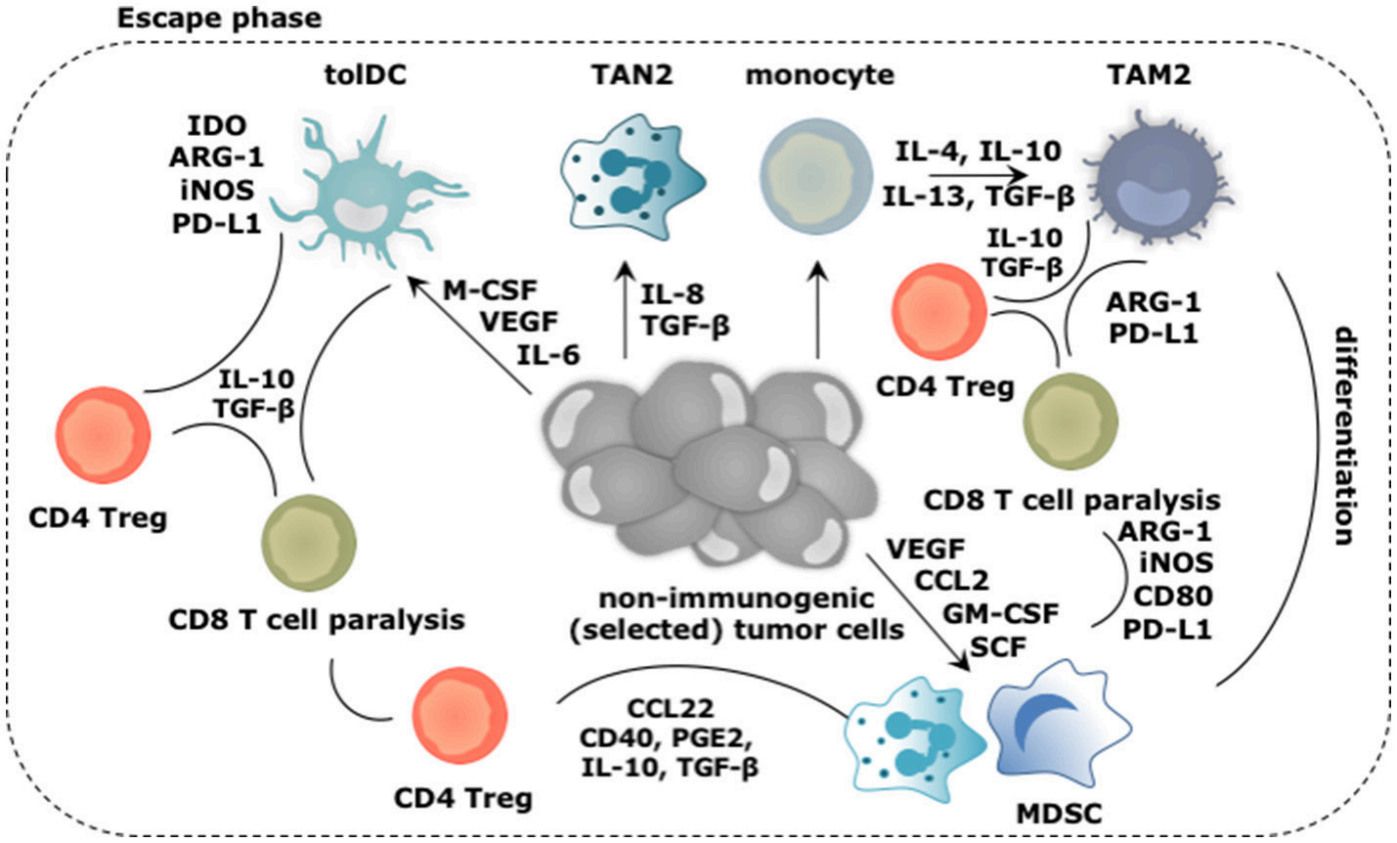
Main roles played by the myeloid cells during the escape of cancer cells from immune mediated destruction. Soluble factors secreted by tumor and immune cells, like CCL2, IL-4, IL-6, IL-8, IL-10, IL-13, GM-CSF, M-CSF, VEGF, and SCF create a TME in which arriving and local myeloid cells are molded into immunosuppressive TIMs. Of these, TAM2, TAN2, and MDSCs are the most abundant, while tolDCs are less frequent in the TME. Cytokines like IL-10 and TGF-β extensively contribute in creating the immunosuppressive TME. These TIMs moreover express a multitude of enzymes (e.g., IDO, ARG-1, iNOS) and surface markers (e.g., CD40, CD80, PD-L1) that support them to dampen antitumor immunity. Moreover, TIMs are not only instructed to suppress antitumor responses under influence of growth factors and cytokines in the TME, they are further instructed to perform activities that are in tune with the needs of the tumor cells. These activities include sustaining chronic inflammation, promoting neo-angiogenesis, tumor growth, invasion and metastasis. These activities are mediated by secretion of soluble factors (e.g., cytokines, growth factors, and proteases). These are not shown in the figure.

Immunoediting is a result of a dynamic process of intricate interactions between immune cells and cancer cells. In this process nascent transformed cells are initially recognized and eliminated by various cells of the innate and adaptive immune system. This continuous immune attack results in selection of transformed cells with non-immunogenic traits, which can evade an immune attack. This heralds the equilibrium phase in which malignant cells co-exist with immune cells. Some of these exert antitumor activities, while others exert tumor-promoting activities. Finally, cancer cells are no longer effectively recognized and killed by the immune system, and thus escape to progress in a full-blown malignant tumor. Myeloid cells have been implicated in each phase of this process (Figures 2, 3).

### The elimination phase

Our knowledge on the events occurring during the elimination phase of the immunoediting process is largely inferred from mouse cancer model studies. These show that an intact lymphoid immune cell compartment is central to the principle of cancer cell elimination. The role of myeloid cells in elimination of nascent transformed cells is less frequently studied with only a limited number of publications on the subject. Nonetheless, several cutting edge studies show that myeloid cells contribute significantly to this stage of the immunoediting process.

Transformed cells can express ligands for the receptor natural killer (NK) group 2 member D (NKG2D), which in mice is expressed on NK cells, CD8^+^ T cells and activated macrophages (26, 27), while in humans its expression is limited to NK cells and CD8^+^ T cells (28). Binding of NKG2D on NK cells sets of its cytolytic functions (29). Other receptors expressed on NK cells that have been implicated in tumor cell recognition and eradication are NKp30, NKp44, and NKp46 (30), and CD226 (DNAM-1) (31). Also loss of MHC-I, which frequently occurs on cancers cells (32), triggers the cytolytic properties of NK cells, as receptors such as Ly49 that provides inhibitory signals to NK cells, are no longer engaged. Studies show that NK cells with reduced Ly49 expression are unable to reject transplanted tumors, suggesting that proper functioning of NK cells is key to tumor rejection *in vivo* (33). Macrophages and DCs can enhance NK cell-mediated cytolytic activity against tumor cells (34, 35). It was established through co-culture experiments that certain tumor cells express N-glycan structures, which can bind dectin-1 on macrophages and DCs, resulting in downstream activation of interferon regulatory factor 5 (IRF5) (35). The activity of IRF5 was critical to induce expression of INAM, a membrane bound protein known to activate NK cells via homophilic interaction (36).

It is further well established that transformed cells express antigens generated due to mutations, epigenetic alterations or aberrant processing of proteins. These tumor-associated antigens (TAAs) are displayed on MHC-I molecules and can be recognized by CD8^+^ T cells (37). These CD8^+^ T cells can receive co-stimulation via triggering of their receptor NKG2D by ligands expressed on the transformed cells (38). It has been further proposed that tumor-rejecting CD8^+^ T cells are stimulated by DCs and macrophages that have taken up fragments of NK cell targeted cancer cells (39). Moreover, cancer cells can express surface calreticulin in response to stress (40). The surface expression of calreticulin serves as an “eat me” signal, allowing DCs to acquire TAAs, which can be cross-presented to CD8^+^ T cells. It was shown that endogenous type I IFN is required to initiate such an antitumor CD8^+^ T cell response, since mice of which the CD8α^+^ DCs lack IFNAR are unable to induce lymphocyte-mediated tumor rejection (41). This endogenous type I IFN could be a result of a DNA damage response, resulting in sensing of cytosolic DNA by the cGAS/STING pathway (42). Next to DCs, macrophages can acquire TAAs by ingesting calreticulin positive cells. As such they could further assist in the activation of CD8^+^ T cells. However, this function of macrophages is counteracted by the expression of SIRPα. This receptor binds to CD47, which is constitutively expressed in various cancer types and serves as a “don't eat me” signal (43).

Another way in which macrophages could contribute to the elimination of nascent cells is through release of nitric oxide (NO) and TNF-α, which in experimental settings can be induced through NK2GD triggering (27). Also other stimuli, which are more relevant to the human setting, such as IFN-γ, anti-CD40 and toll-like receptor (TLR) ligands, can induce the release of NO and TNF-α, thereby stimulating the tumoricidal activity of macrophages (44). At least for IFN-γ, there are studies that suggest this might occur during the early stages of cancer formation. It was shown in a model of MHC-II negative multiple myeloma that macrophages are rapidly recruited and present TAAs to CD4^+^ T cells, which in turn activate the tumoricidal program in macrophages through secretion of IFN-γ (45). Also NK cells and CD8^+^ T cells produce high levels of IFN-γ, thereby provoking the hypothesis that also these lymphocytes can stimulate the tumoricidal properties of macrophages.

### The equilibrium phase

The coordinated immune attack, executed during the elimination phase, can annihilate transformed cells. However, continued pressure of the immune system can result in selection of cancer cells that can prevail. Cancer cells can avoid immune recognition in several ways, among which downregulation of antigen processing machinery, or expression of TAAs or MHC-I molecules. Consequently, cancer cells with reduced immunogenicity are selected for. Several preclinical studies have provided evidence for a role of CD8^+^ T cells in this process (46). The clinical relevance hereof was suggested in several cancer trials, showing selective downregulation of the targeted TAA or MHC-I in recurring tumors (47–49). Also myeloid cells were shown to play a role in shaping the immunogenicity of tumors. By educating CD8^+^ T cells, they contribute indirectly to the tumorigenicity. A more direct action was suggested for TAM1 (50). It was shown that in the in absence of T cells, NK cells can activate TAM1 through the production of IFN-γ, and that these TAM1 served as modulators of the tumor's immunogenicity. In addition to camouflaging themselves, cancer cells co-opt regulatory circuits to dampen immune responses. These include but are not limited to expression of PD-L1, recruitment of regulatory T cells (Tregs), and regulatory myeloid cells. The many mechanisms exploited by regulatory cells to paralyze tumor-specific effector cells and as such create a tolerant environment for cancer cells has been reviewed elsewhere (51, 52). Consequently, tumor clones survive the elimination checkpoint and form a clinically occult tumor that co-exists with host immune cells. During this dynamic equilibrium, immune cells with anti- and protumor activities can be found in the vicinity of the tumor cells (53).

Cell death coinciding with the presence of surface calreticulin and alarm signals such as type I IFN is key to antitumor immune activation by DCs (41). This type of cell death, when further associated with release of adenosine triphosphate (ATP), high mobility group box 1 (HMGB1) proteins, and annexin A1 (ANXA1), has been coined immunogenic cell death (ICD). While cell death due to ROS in combination with endoplasmic reticulum (ER) stress leads to ICD in tumor cells, apoptosis induced by exposure of tumor cells to IFN-γ and TNF-α is less immunogenic (54). This can induce quiescence, implicated in tumor dormancy (55, 56). Also, the pro-inflammatory signals that HMGB1 normally triggers on myeloid cells are strongly diminished when this protein gets oxidized (57). Moreover prolonged production of ROS and RNS by myeloid cells can increase the events of DNA mutation in tumor cells, and as such be a cause of cancer progression in itself (58). Several mechanisms are built into the inflammatory cascade that occurs during the elimination phase, to prevent excessive collateral tissue damage and auto-immunity. For example, debris clearance through phagocytosis and autophagy results in secretion of TGF-β, which functions as a strong immunosuppressant and activates type 2 polarized TIMs, resulting in CXCL5-mediated inflammation and tumor growth (25, 59). Even T_H_1-linked cytokines can help in the accumulation of TIMs with regulatory functions. Both IFN-γ and IL-1β were shown to participate in the mobilization, recruitment, and inhibitory activity of MDSCs (60). Transcription factors that govern these events shift from pro-inflammatory IRF8 and signal transducer and activator of transcription (STAT) 1, 2, 3, and 6 to CCAAT-enhancer-binding protein β (C/EBPβ) and PI3Kγ, generating immature TIMs. Moreover glycolysis, the metabolic pathway preferred by TIMs and tumor cells during the elimination phase, mediates production of proteins, nucleic acids, lipids, lactate, adenosine, and consequently immunosuppressive acidification of the TME. As a result TIMs undergo peroxisome proliferator-activated receptor gamma (PPARγ) and STAT-mediated metabolic reprogramming (61). They switch from glycolysis to oxidative phosphorylation and lipid catabolism (fatty acid oxidation). Inhibition of the glycolysis pathway in TAN1, TAM1, and mature DCs shortcuts their activities, thereby favoring the activity of tumor-promoting TIMs, eventually leading to the escape phase, where immunologically sculpted tumors grow progressively, become clinically apparent and have an established immunosuppressive TME (62).

### The escape phase

The early immune response to cancer cells is purposed to deal with the danger at hand and is characterized by acute inflammation. However, persistence of cancer cells coinciding with the co-existence of immune cells with pro- and antitumor properties results in smoldering inflammation. This chronic inflammation is mainly sustained by TAM2, TAN2, and MDSCs, which are the main TIM subsets found within many established cancers. This is not surprising as tumor cells produce factors such as G-CSF, GM-CSF, and VEGF to ensure continuous recruitment of myeloid progenitors into the circulation. Under physiological conditions, GM-CSF drives myelopoiesis as well as DC differentiation, while G-CSF and M-CSF establish the differentiation, proliferation and survival of granulocytes and macrophages respectively (10). In chronic inflammation however, GM-CSF downregulates IRF8 in DC progenitors, and thus results in reduced DC development next to recruitment of more MDSCs. Furthermore, M-CSF suppresses the differentiation of DCs while enhancing TAM2 polarization (63). Therefore, this factor has been attributed as a predictor of poor prognosis (64). G-CSF can mobilize granulocytic myeloid cells from the bone marrow to promote angiogenesis. Moreover, immature TIMs will drive the recruitment of more regulatory TIMs resulting in a “never-healing-wound.” Factors that aid in the recruitment of myeloid cells, such as monocytes, DCs, MDSCs, and TAN2 to the TME, are among others IL-4, IL-6, IL-8, IL-10, S100A8/A9, VEGF, and TGF-β (65).

The tumor promoting functions of TIMs are typically activated by TLR triggering and binding of cytokines and growth factors such as IFN-γ, IL-4, IL-6, IL-10, IL-13, and TGF-β to their responding receptor. The signaling cascade following receptor engagement results in upregulation of various transcription factors, including STAT1, 3 and 6, mitogen activated protein kinase-extracellular regulated kinase (MAPK-ERK) and nuclear factor-κB (NF-κB), with STAT3 being a master regulator in most myeloid cell subsets (66–68). Once activated, TIMs perform overlapping functions. TAM2 and MDSCs are well known for their ability to suppress T cell-mediated tumor killing (Figure 3) since both can exert direct immunosuppressive effects using cell-cell contact (e.g., CD80 and PD-L1), secreted factors (e.g., IL-10 and TGF-β) and expression of enzymes (e.g., ARG-1, iNOS, IDO) (69–75). In addition, TAM2, and MDSCs engage Tregs to aid them in suppressing antitumor immune responses. To that end, these TIMs express and produce several factors that stimulate the differentiation (e.g., IDO, CD40, prostaglandin E2 [PGE2], IL-10, TGF-β), or recruitment of Tregs (e.g., CCL22) (76–78). TAM2 can further interfere with the activity of TAM1, suppressing the expression of IFN-γ and IL-12, thereby impacting on direct tumor cell killing as well as the activation of killer T cells (65). Although TAN2 are not notorious for their T cell suppressive activity, it has been shown that eliminating TAN2 results in increased cytotoxic T cell activity, a phenomenon that was linked to TGF-β, which favors TAN2 accumulation and induces ARG-1 expression (16, 79). Although DCs are not abundantly present in the established TME, it is essential to discuss their role in facilitating the escape of tumor cells from immune killing (Figure 3). Conditions in the TME like accumulation of extracellular adenosine and lactate, VEGF, M-CSF, TGF-β, IL-6, and hypoxia, act in concert to inhibit antigen presentation by DCs, therefore inhibit activation of adaptive antitumor immunity (80–82). Moreover during tumor progression, subsets of DCs are able to reach tumor-draining lymph nodes and stimulate proliferation of Tregs in a TGF-β dependent way, turning immature DCs from a victim to a partner in crime (83). It has even been described that mature DCs within the TME can exert protumor properties through expression of IDO, ARG-1, and iNOS. These mature but tolDCs block the proliferation of T cells and in the presence of TGF-β further stimulate Treg differentiation (84–87).

Promoting angiogenesis by secreting VEGF, angiopoietin 1, and 2 and GM-CSF, and tumor cell dissemination by secreting MMPs and cathepsins are two other features shared between TAN2, tolDCs, TAM2, and MDSCs (88). Moreover, it has been proposed that TIMs like MDSCs and TAN2 are responsible for creating a pre-metastatic niche (89–93). In these pre-metastatic niches, factors are produced to attract and later on arrest tumor cells such as IL-1β, prokineticin 2, and MMPs while neighboring cells are stimulated to secrete VEGF to facilitate the arrival of tumor cells.

In conclusion, the TME instructs TIMs to perform functions in tune with the tumor's needs. These functions are related to chronic inflammation, tissue remodeling and immunosuppression, and promote tumor growth and metastasis, as these functions enable tumor cells to acquire nutrients, proliferate, survive, migrate, and escape immune elimination.

## Cancer therapies and myeloid cells: mutual influence and implications

**Figure 4:**
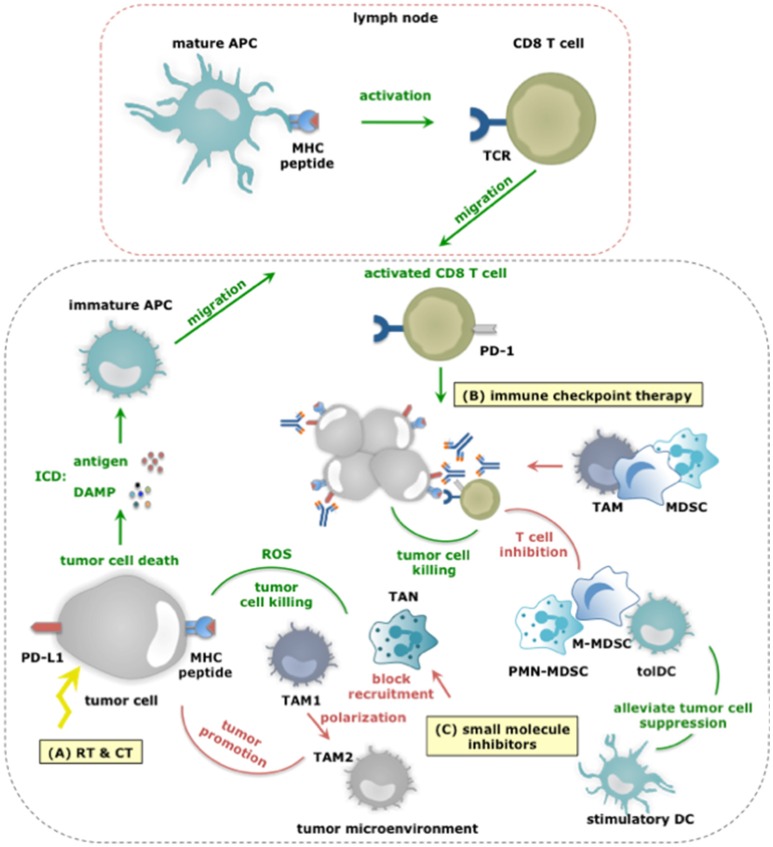
The dual role of myeloid cells in anticancer therapy responses. **(A)** RT and CT regimens can induce ICD, thereby alarming antigen-presenting cells (APCs) of the danger that is posed by cancer cells. This alarm is given through the release of various DAMPs (e.g., HMGB1, ATP, et cetera) and facilitates cross priming of tumor-specific CD8^+^ T cells in tumor draining lymph nodes. Activated CD8^+^ T cells subsequently infiltrate the tumor in search of TAA-expressing cancer cells, and upon recognition exploit various mechanisms to kill cancer cells. While, TAM1 and TANs can further enhance the efficacy of CT by releasing ROS, thereby enhancing tumor cell death, other TIMs including MDSCs can counteract the effects or RT and CT not in the least by inhibiting CD8^+^ T cells. **(B)** Within the TME, CD8^+^ T cells are often rendered tolerant via immunosuppressive factors expressed by tolDCs, TAM2, MDSCs, tumor cells (and Tregs). One strategy is expression of PD-L1 that binds to PD-1 on activated T cells. Consequently, immune checkpoint inhibition has been studied to alleviate PD-L1:PD-1 mediated immunosuppression. Monoclonal antibodies are actively used for this purpose, however, were shown to be captured by TAMs using their FcγR and were shown to be counteracted by MDSCs. Nonetheless, the success of immune checkpoint inhibition is correlated to the presence of immunostimulatory TIMs and CD8^+^ T cells within the TME. **(C)** Small molecule inhibitors targeting protein kinases implicated in tumor cell progression have been shown to exert effects on TIMs. Some of these effects are beneficial for therapy outcome. For instance BRAF inhibitors revert the suppression exerted by melanoma cells on TADCs. However the majority of these effects potentiate TIMs to exert tumor-promoting functions or prevent TIMs from entering the TME to oppose tumor growth. Examples hereof are Imatinib that stimulates TAM1 to TAM2 polarization, BRAF inhibitors that restore the MDSC compartment through induction of CCL2 and MET inhibitors that avoid recruitment of neutrophils with cytotoxic capactity (iNOS mediated NO release).

Experimental studies have provided ample evidence that myeloid cells influence virtually every type of cancer therapy, ranging from chemotherapy (CT), radiotherapy (RT), tumor targeted therapy to immunotherapy (Figure 4). Therefore, the following sections will briefly discuss the interplay with various cancer therapies.

### Myeloid cells and radiotherapy

Radiotherapy (RT) uses ionizing radiation to kill malignant cells and can induce full-blown ICD in a dose-dependent manner (94). ICD allows TADCs to acquire TAAs as well as stimuli that enforce their maturation, therefore enable them to activate tumor-specific CD8^+^ T cells (95, 96). Moreover, low doses of RT can reprogram TAMs into TAM1, which can contribute to the recruitment and activation of tumor-specific T cells (97). However, RT-mediated immunogenic effects can be masked (98, 99). In an established TME, TIMs are instructed to counteract antitumor immune responses. Depending on the dose, its fractionation and also the tumor model, RT can recruit myeloid cells and even polarize them into tumor-promoting immunosuppressive cells (100). The latter finding suggests that depletion of TIMs could be a strategy to enhance the outcome of RT. It was indeed shown in several experimental studies that depletion of TAMs, MDSCs and neutrophils enhances the efficacy of RT (101–103). Caution must however be taken to ensure that RT-mediated effects on TADCs and TAM1 polarization are not lost, as both contribute to the abscopal effects of RT.

### Myeloid cells and chemotherapy

Chemotherapy (CT) comes in many colors and shapes. Similar to RT certain CT regimens, like use of anthracyclines, can induce full-blown ICD, thereby acting on TADCs, which in turn activate tumor-specific CD8^+^ T cells. The efficacy of anthracyclines was shown to depend on TADCs (104, 105). Also TAMs can play an advantageous role during CT treatment. These cells can act as drug depots when CT compounds are delivered in nanoparticles, thereby ensuring local and prolonged delivery of the therapy (106). Moreover, ROS production by TAMs and TANs is observed upon treatment with oxaliplatin, and aids in stimulating tumor cell death (107). Chemotherapies can further act on TIM numbers, either depleting or amplifying them. Gemcitabine and 5-fluorouracil are examples of chemotherapies that reduce MDSC numbers. By relieving MDSC-mediated immunosuppression these treatments enable CD8^+^ T cell-dependent anticancer immune responses (108). Other chemotherapeutics, like paclitaxel show a more complicated profile. While paclitaxel depletes MDSCs (which express P450 reductase), it increases TAMs (12, 109). In case of the latter, inhibiting TAM accumulation enhanced therapy outcome (110). This study suggests that TAMs can hamper the efficacy of CT. This is confirmed by several other studies, and unfortunately is not limited to TAMs. Also MDSCs have been linked to CT failure. TAMs and MDSCs can induce drug resistance for instance by producing cysteine cathepsins that protect tumor cells from being killed by chemotherapeutic agents like taxol, and even promote tumor growth by enforcing chronic inflammation (111, 112). Moreover, TAMs can secrete IL-10 in response to CT and as such enforce immunosuppression (113). Thus, TIMs are both friend and foe when treating cancer patients with chemotherapies (and RT), posing the challenge to identify and selectively act on the enemy, while leaving friendly TIMs unharmed.

### Myeloid cells and tumor targeting small-molecule cancer therapy

Our growing knowledge on driver mutations that provide cancer cells with a growth and survival advantage has allowed the development of small-molecule drugs that target the molecular alterations arising from these mutations. Proteins with kinase activity such as stem cell factor receptor (c-kit), BRAF, MEK, and MET are examples of targets for which small-molecule inhibitors were clinically tested. In clinical trials these small-molecule inhibitors initially reduce the tumor size (114, 115). However, they have little effect on the long haul, which might at least in part be due to activities of myeloid cells. For instance Imatinib, a tyrosine kinase inhibitor that works via activating mutations of c-kit, has been evaluated in gastrointestinal cancer. Imatinib was shown to induce tumor cell apoptosis. While this is the envisaged outcome, it was further observed that TAM1, which mainly populated the gastrointestinal stroma were polarized to TAM2 due to activation of C/EBPβ upon interaction with dying tumor cells (116). BRAF and MEK are protein kinases of the MAPK-ERK pathway, which in solid tumors such as melanoma enable proliferation and cell survival. Consequently, mutations of BRAF and MEK, which continuously activate MAPK-ERK are linked to oncogenesis. Small-drug inhibitors were developed to counteract the uncontrolled growth and survival of cancer cells through BRAF and MEK mutations (117–120). Development of resistance to BRAF inhibitors in a mouse melanoma model was linked to restoration of the MDSC compartment. This depended on reactivation of the MAPK pathway followed by production of CCL2, a myeloid cell attractant. Modulating MDSCs using depleting antibodies (anti-Gr-1) or blocking their recruitment (CCR2 antagonist) prohibited the outgrowth of these BRAF resistant melanoma tumors (121). Also TAMs and TAM-derived TNF-α have been implicated in failure of BRAF inhibitors in mouse melanoma models (122). Blocking TAM development (inhibition M-CSFR) improved the efficacy of BRAF inhibitors (123). On the other hand, MEK, and BRAF inhibitors revert TADC suppression enforced by mutated melanoma cells *in vitro* (124, 125). Finally MET (hepatocyte growth factor receptor) is considered a molecular target in several cancers, including non-small cell lung cancer, gastrointestinal cancer, and hepatocellular carcinoma (114). Inhibition of MET also impacts on neutrophils, which rely on MET activity to home to tumors and exert cytotoxic responses such as iNOS-mediated release of NO (126). The above studies show that further studies are required to understand how inhibiting kinases impact on myeloid cells and how myeloid cells can be manipulated to allow more durable responses in patients.

### Myeloid cells and immune checkpoint therapy

Immune activation and activity are tightly regulated by several mechanisms to avoid immunopathology (e.g., auto-immunity). Cancer cells hijack these mechanisms to escape immunosurveillance. They express ligands, such as PD-L1, which triggers PD-1, an inhibitory receptor expressed on tumor-reactive immune cells, such as CD8^+^ T cells. Consequently, the tumor-reactive immune cells become paralyzed. Monoclonal antibodies (mAbs) that bind PD-1 or PD-L1 and block this inhibitory immune checkpoint have ushered a new era for cancer immunotherapy (127–129). To date, five mAbs that target the PD-1:PD-L1 pathway have been approved for treatment of patients with solid tumors, including melanoma and non-small cell lung cancer. These mAbs are pembrolizumab and nivolumab, which antagonize PD-1, and avelumab, darvulumab and atezolizumab, which antagonize PD-L1. These mAbs have signified a turning point in the treatment of a significant number of patients (130). Regrettably the full potential of these mAbs has not yet been achieved.

A considerable number of patients and cancers are refractory at the start of treatment or become resistant during the course of treatment and therefore fail the therapy. Several hypotheses exist to explain this therapy failure, one of them being that TIMs are responsible for the resistance to immune checkpoint blocking therapy. This hypothesis resulted from the observation that high infiltration of tumors with immunosuppressive myeloid cells correlates with poor prognosis and immune checkpoint therapy resistance (131, 132). Mainly MDSCs and TAMs have been implicated in resistance to immune checkpoint blocking mAbs. Therefore, merely blocking PD-1:PD-L1 interactions might be insufficient to overcome TIM-mediated inhibition of CD8^+^ T cells. A line of thought that is reinforced by the observation that MDSC depletion in experimental models was shown to enhance antitumor immune responses and overcome resistance (133). Moreover, functional modulation of MDSCs by epigenetic drugs, sensitized several experimental cancer models that were previously resistant to immune checkpoint therapy (134, 135). Further TAMs have been described to inhibit PD-1:PD-L1 therapy by removal of anti-PD-1 mAbs from PD-1^+^ CD8^+^ T cells using their FcγR (136). Additionally emerging data report that both metabolic and inflammatory pathways can enhance the expression of PD-L1 on myeloid cells (71, 137, 138). These studies provide the first proof that immunosuppessive TIMs can hamper immune checkpoint therapy via several redundant strategies. However, there is also proof that immunostimulatory TIMs are a pre-requisite for immune checkpoint success. It was shown that binding of monocytes and DCs by anti-PD-L1 mAbs is at least in part responsible for the subsequent control of tumor growth (139, 140). The need for DCs to guarantee successful checkpoint therapy has further been evidenced in experiments, in which delivery of bone marrow-derived DCs to tumors resulted in T cell recruitment and sensitization to anti-PD-1 therapy (141). Similar to the conclusion on myeloid cells in RT, CT and targeted therapies, we can conclude that immune checkpoint therapy can be supported by TIM allies, i.e. TIMs that can activate CD8^+^ T cells, while immunosuppressive TIMs that exert several means to paralyze CD8^+^ T cells, can be regarded as the saboteurs of this therapy. Besides the PD-1:PD-L1 pathway, other checkpoint inhibiting receptors or their ligands have been reported to be expressed by one or more TIM subsets. For instance, the expression of T-cell immunoglobulin and mucin-domain containing-3 (TIM-3) has been described in TADCs while ‘V-domain Ig suppressor of T cell activation’ (VISTA) is predominantly expressed on hematopoietic cells, and in multiple murine cancer models found at particularly high levels on TIMs. Further the “T cell immunoreceptor with Ig and ITIM domains” (TIGIT) has been reported to bind CD155 on DCs and macrophages while the main ligand of “Lymphocyte-activation gene 3” (LAG-3) is represented by MHC-II, expressed in various degrees on almost all TIMs. Future investigation is therefore warranted to find out which role TIMs play in other checkpoint inhibiting mechanisms.

## Strategies developed to target myeloid cells in order to resolve cancer

**Figure 5:**
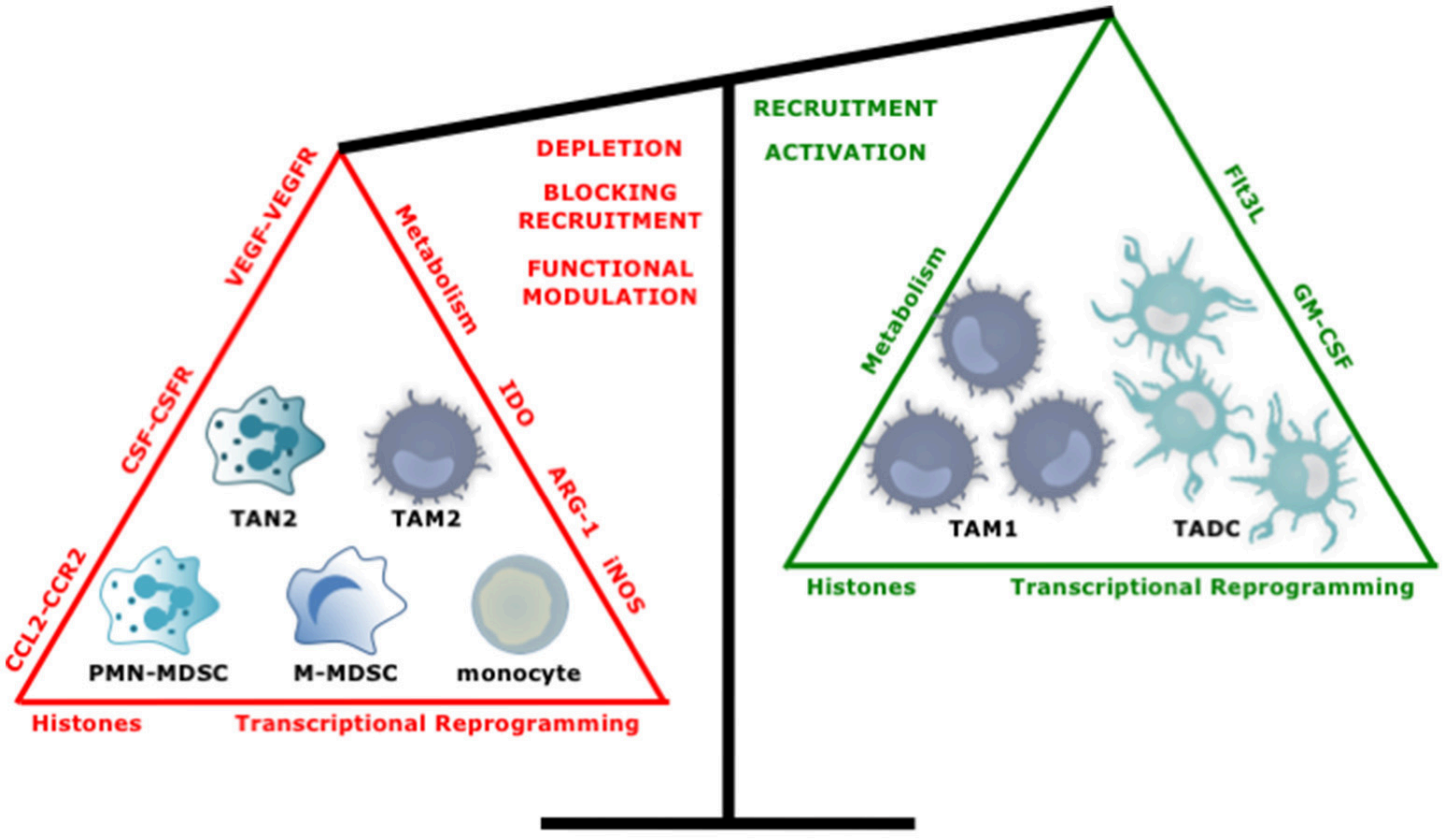
Tipping the balance toward myeloid cells with an antitumor phenotype. Several approaches have been studied to increase the ratio of anti- over protumor TIMs. These include, depleting or repolarizing tumor-promoting TIMs, and attracting and activating antitumor TIMs.

An increasing number of experimental and clinical studies have been published on targeting of myeloid cells in cancer with close to 400 studies published in the last 5 years. Overall the approaches to target myeloid cells with tumor-promoting characteristics like TAM2, TAN2, and MDSCs can be divided into two main strategies; reducing their numbers by depleting them or by blocking their recruitment, and repolarization of their function. Conversely, attempts have been made to enhance the numbers and function of myeloid cells with antitumor properties. In the following sections we highlight several promising strategies (Figure 5).

### Manipulating myeloid cell numbers

Depleting myeloid cells and even specific subsets, as a strategy to reduce myeloid cell numbers, can be performed using antibodies that target myeloid cell specific surface makers such as CD11b, Gr-1 or Ly6G. Furthermore also transgenic mouse strains can be used with permanent or conditional myeloid cell ablation. Conditional deletion circumvents embryonic lethality while precluding analysis of gene function on a well-defined time point in the adult tissues. The two most commonly used models are based on the addition of diphtheria toxin and/or Cre/LoxP recombinase. The former model is based on a knock-in of the diphtheria toxin receptor behind the promotor of a cell type characterizing marker such as CD11b or CD11c-diphteria toxin receptor mice. In contrast, the generation of conditional Cre/LoxP knockout mice is a multiple-step process, which involves mating flox mutant with tissue specific Cre-expressing mouse lines. As such the latter allows conditional myeloid cell subtype specific ablation in a particular tissue of interest.

Such experimental approaches have delivered a proof-of-concept that depletion of myeloid cells with immunosuppressive functions can delay tumor growth, while depleting stimulatory myeloid cells has the opposite effect (142, 143). As these approaches do not limit myeloid cell depletion to the TME, it is however not certain that the observed effects can in part be ascribed to depletion of myeloid cells in the periphery. Moreover, complete depletion strategies are not attractive for translation to a human setting. Therefore, other pharmacological and gene-based approaches have been developed to interfere with the accumulation of suppressive myeloid cells. These are based on our current knowledge on how TAM, MDSC and TAN myelopoiesis and recruitment are driven. Accumulation of TAMs in the TME depend strongly on CCL2:CCR2 and M-CSF:M-CSFR signaling, therefore different approaches using mAbs (144–146), small-molecule inhibitors (145, 147, 148) and RNA interference (149) were devised to block these pathways. Overall these studies showed reduced TAM numbers and correlating herewith, delayed tumor outgrowth in various mouse cancer models. These studies further provided insight into the effect of CCL2:CCR2 and M-CSF:M-CSFR blockade, and how treatment regimens might evoke unwanted effects.

When blockade of the CCL2:CCR2 axis was halted in mice, a sudden burst of monocytes released from the bone marrow was observed, and an increased frequency of metastasis and linked herewith decreased survival of animals was the consequence (110). Use of CCL2 versus CCR2 knockout mice further revealed that deficiency in CCL2 resulted in delayed outgrowth of mammary carcinoma, while deficiency in CCR2 enhanced tumor outgrowth (150). Differences in transcriptional programs in monocytes deficient in CCR2 vs. CCL2 might explain these disparate effects of CCR2 vs. CCL2 disruption on progression of mammary carcinoma. While this study did not show differences in monocyte or TAM infiltration, it is conceivable that other TIM subsets might play a role. For instance, also MDSCs can act on CCL2 through their expression of CXCR2 (151). Similar to interfering with the CCL2:CCR2 axis and TAM accumulation, interfering with both the CCR2^+^ TAMs and CXCR2^+^ TANs has profound effects at least when used in combination with CT (152, 153). It is therefore of utmost importance to identify all relevant players when blocking a certain molecule in order to fully understand the potential strengths and weaknesses of the depletion approach.

Targeting the M-CSF:M-CSFR pathway is also interfere with TAM accumulation. This approach delays tumor growth in all experimental models tested, either when used as a stand-alone therapy (146, 147), or when used in combination with other therapies (101, 102, 109, 113). Certain studies in which the M-CSF:M-CSFR pathway was targeted reported on tumor regression, which was not linked to numerical changes in TAMs in the TME. However, changes in the TAM phenotype, particularly downregulation of TAM2 associated features, were reported in these studies (145, 149). Because interrupting CCL2:CCR2 and M-CSF:M-CSFR signaling has been beneficial in many experimental models, these pathway are being evaluated in patients both as mono- (146) and combination therapies (CT and immune checkpoint blocking therapy) (154).

Another strategy that is explored in the context of reducing tumor-promoting myeloid cell numbers in the TME is interfering with VEGF:VEGFR signaling. VEGFR is highly expressed by TIMs and interaction with VEGF contributes to their migration and differentiation into immunosuppressive cells (155). Thus, interfering with VEGF:VEGFR signaling might have a dual role on TIMs, reducing their numbers and repolarizing their function (in addition to affecting angiogenesis). In view of attracting TIMs, VEGF:VEGFR signaling was shown to attract macrophages (156) and monocytes in a CCR2-dependent way (157). It was further shown that also recruitment of MDSCs to the TME of non-small cell lung carcinoma is reduced upon blockade of VEGF:VEGFR signaling. However, recruitment of monocytes increased upon treatment with anti-VEGF antibodies in this study (158). Several anti-VEGF-based drugs, including bevacizumab, avastin and axitinib, targeting VEGFR are used for treatment of various human cancers (159–161). While the anti-VEGFR targeting drugs used in these studies are mainly studied in view of angiogenesis, it was shown at least for the drug axitinib that it impacts on myeloid cells. A dose-dependent reduced activation of STAT3 was observed in axitinib-exposed DCs. Despite this favorable change, these DCs were impaired in their T cell stimulatory activity (162). In contrast, it was shown in a model of melanoma brain metastasis that axitinib increases the number of M-MDSCs, and reverts their function from T cell suppressive to stimulating cells with a subsequent delay in disease progression (163). Therefore, this study suggests that enriching myeloid cells with antitumor properties might be an attractive approach to treat cancer. This idea is supported by experimental and clinical studies in which *ex vivo* DCs (often engineered to secrete stimulatory cytokines), delivered to the tumor, mediate disease control (164, 165).

The use of Flt3-L to enhance DC numbers has been extensively studied, since Flt3:Flt3-L mediated signaling is involved in the generation of various DC subsets from bone marrow progenitor cells (166, 167). In experimental models, delivery of Flt3-L (alone or in combination with CT) increased DC numbers and resulted in delayed tumor outgrowth (168–171). Evidence supporting the use of Flt3-L in a clinical setting came from a study in which recombinant human Flt3-L was administered to expand DCs *in vivo*. These DCs were collected by leukapheresis, pulsed with tumor antigen-derived peptides and used for vaccination, eliciting tumor regression in 2 out of 12 patients (172).

Also GM-CSF has been studied as a means to enhance DC numbers *in vivo*. This might seem controversial given the role of GM-CSF in myelopoiesis. It is certainly a strategy that has to be approached with caution, as blocking GM-CSF has been shown to reduce accumulation of monocytes and myeloid precursor cells, and was shown to delay tumor progression, while delivering GM-CSF to tumors, for instance using the oncolytic virus T-VEC, was shown to induce tumor-specific T cells, most likely as a consequence of DC activation. In this clinical study, delivery of GM-CSF by T-VEC also resulted in a reduced number of MDSCs (173, 174). The latter is surprising as engineering of tumors to express high levels of GM-CSF has been linked to MDSC accumulation in various experimental models (175, 176).

Overall these studies support the idea that decreasing the number of TIMs with tumor-promoting activities, while increasing TIMs with antitumor activities, is a valuable approach in the fight against cancer. These studies however also point out that tipping the balance toward increased numbers of antitumor TIMs by acting on pathways involved in myelopoiesis is a challenging endeavor.

### Manipulating the function of myeloid cells

Myeloid cells are characterized by high plasticity. Their ability to shift shape according to the cues they receive, has instigated research into strategies that promote their anti- and/or inhibit their protumor activities.

#### Manipulating genetic programs in myeloid cells

Genetic programs, controlled by transcriptional networks, and epigenetic mechanisms, dictate the phenotype, and function of myeloid cells. Therefore, reprogramming them has been a focus of interest. Triggering TLRs on TIMs, in particular TLR3 and TLR9, has been extensively studied in this context, as these jumpstart transcriptional networks. It was shown that the T cell suppressive activity of MDSCs is reduced when exposed to agonists of TLR3 or TLR9 (177–180). Exposing MDSCs to the TLR9 ligand CpG stimulated them to produce T_H_1 activating cytokines and even differentiate into TAM1, a switch that was driven by IFN-α, produced by plasmacytoid DCs after CpG stimulation (178, 181). Besides its effect on MDSCs, TLR3 triggering also converts TAM2 to TAM1 and facilitates cross-priming of antigen-specific CD8^+^ T cells by TADCs, thereby promoting tumor regression (182, 183).

The use of TLR4 to functionally reprogram DCs was achieved by a DC unique genetic approach, which is likely explanatory for its success, as it was previously shown that inflammation primed MDSCs are activated by lipopolysaccharide (LPS), a well-known TLR4 ligand, to produce increased levels of IL-10 and to downregulate production of IL-12 by macrophages (184).

The CD40:CD40 ligand (CD40L) interaction may represent another interesting target to dampen the protumor transcriptional program of MDSCs, while stimulating the antitumor transcriptional program of TAMs and TADCs. Agonistic CD40 mAbs were shown to abrogate MDSC:Treg communication and to mature MDSCs (185, 186). Macrophages are activated upon treatment with these CD40 mAbs, enabling them to infiltrate tumors and exert tumoricidal activities (187). The observation that anti-CD40 mAbs enable CD8^+^ T cell independent tumor cell killing, prompted the use of a fully humanized CD40 agonist mAb (CP-870.893) in a phase I clinical trial, showing that its combination with gemcitabine activates antitumor immune responses (188). Activation of DCs by CD40 licenses them to perform their CD8^+^ T cell stimulatory function (189). Combining CD40 and TLR3 ligands was shown to endow human and mouse tolDCs with stimulatory properties (190). Also TLR4 in addition to CD70 delivery was studied in combination with CD40 activation of TADCs (191). It was shown that TADCs modified with CD70 and simultaneously activated via CD40 and TLR4, were able to migrate to tumor draining lymph nodes and stimulate tumor-specific CD8^+^ T cells.

Cytokines represent another means of functionally reprogramming myeloid cells. In this context, IL-12 and type I IFNs have been shown to stimulate antitumor properties, while IL-10 and TGF-β have been linked to tumor-promoting properties. Consequently, exposing TIMs to IL-12, IFN-α or IFN-β, and/or shielding them from IL-10 and TGF-β has been studied. Delivery of IL-12 to the TME was shown to have substantial antitumor effects, which were correlated to reprogramming of MDSCs, TADCs and TAMs into antigen presenting cells with CD8^+^ T cell activating capacity (175, 192–196). The antitumor stimulating properties of IL-12 were confirmed by the clinical responses observed in patients with renal cell carcinoma, melanoma and peritoneal metastasis from ovarian cancer upon IL-12 treatment (197–202). Type I IFNs, in particular IFN-α and IFN-β, have long been shown to exert tumoricidal effects and act as strong inducers of myeloid cell polarization, mainly for MDSCs and TADCs (203, 204). Although IFN-α has shown promising clinical results in hematopoietic cancers, little success was achieved in solid tumors (205). This might be related to its systemic administration or its use as a single agent in these trials. It was shown in preclinical studies that combining IFN-β stimulation of myeloid cells with simultaneous blockade of TGF-β signaling, is a viable strategy to reprogram MDSCs and DCs, thereby facilitating CD8^+^ T cell responses (206). Also IL-10 switches on immunosuppressive transcriptional programs in myeloid cells. IL-10 receptor blocking antibodies, used alone or in combination with CpG, restored IL-12 expression through activation of DCs, and stimulated antitumor responses (113). However, in some studies ablating IL-10 was associated with an increased level of MDSCs and Tregs in TME and in the tumor-draining lymph nodes (207).

Strategies to manipulate the transcriptional programs in myeloid cells more directly have been developed as well, including approaches to down- or upregulate transcription factors like STAT3 and NF-κB (208, 209). Transcription factor STAT3 has been designated the master regulator of the immunosuppressive activity of myeloid cells (66, 67). Studies with small-molecule inhibitors, acting on JAK2:STAT3 signaling, showed delayed tumor growth, which in some cases coincided with a TAM2 to TAM1 switch, an increase of MDSCs or was independent of TIM modulation (210, 211). In clinical trials, small molecular inhibitors showed limited efficacy and substantial side effects, prompting the development of more targeted and more specific strategies based on RNA interference and decoy oligonucleotides. Some of these entered clinical testing (212–214). More selective delivery of STAT3 inhibiting small interfering RNA (siRNA) was achieved by coupling them to CpG. Therefore, this strategy takes advantage of the positive effects of CpG on TLR9 positive myeloid cells as well as the potential effects of STAT3 inhibition. Upon treatment, TLR9 expressing myeloid cells like PMN-MDSCs, showed reduced immunosuppressive capacity. Also TLR9 expressing tumor cells lost the resistance to apoptosis by interfering with STAT3 signaling (212, 213, 215, 216). The less activated status of TADCs and their overall reduced ability to respond to TLR stimulation has been related with STAT3 hyperactivity (215). Therefore, STAT3 inhibition has been studied in the context of TADC modulation. Herein, advantage was taken of the TADCs' ability to take up nanoparticles to deliver STAT3 siRNA and polyinosinic:polycytidylic acid (polyI:C), a well-known agonist of TLR3. This strategy enabled TADCs to upregulate the expression of co-stimulatory molecules and IL-12, and to induce potent antitumor immune responses (215). Next to STAT3, NF-κB is known to regulate the expression of many tumor-promoting genes, including VEGF, IL-6, TNF-α, and cyclooxygenase 2 (COX2), which support its crucial role in the activation of TAM2 in the TME (217, 218). Inhibition of NF-κB allows TAMs to acquire a tumoricidal phenotype, resulting *in vivo* in regression of advanced tumors (219). In TADCs, NF-κB activity is inhibited by FOXO3, which has been related to the TADCs' immunosuppressive activity (220). Activation of NF-κB in DCs has been extensively studied albeit mainly in the context of cancer vaccination. Overall these studies showed that selective activation of NF-κB in DCs of human or mouse origin enhances their ability to activate CD8^+^ T cells (209, 221). Another transcription factor that recently gained attention is PI3Kγ, as this transcription factor was shown to govern expression of ARG-1 and TGF-β in TAMs, therefore represents another important regulator of the immune suppressive phenotype of TAMs (222). Furthermore, PI3Kγ in myeloid cells has been directly linked to the success of immune checkpoint therapy, pinpointing PI3Kγ as an attractive target in cancer immunotherapy (222, 223).

The genetic programs active in myeloid cells are also regulated at the epigenetic level (138). Alternatively activated bone marrow-derived macrophages have enhanced levels of acetylated histones. This was linked to expression of TAM2 genes (224). Bromodomain and extra-terminal motif proteins (225) or histone deacetylase inhibitors (HDACi) (226–228), which interfere with chromatin remodeling, skew toward a TAM1 phenotype and were shown to mediate tumor regression in response to immune checkpoint therapy in a model in which monotherapy with anti-PD1 antibodies failed (228). Also MDSCs were shown to be numerically and functionally affected by HDACi (134, 135). Inhibition of HDAC reduced the number of MDSCs as well as the expression of hallmark enzymes, including ARG-1 and iNOS, making several experimental cancer models sensitive to immune checkpoint therapy (134, 135). HDACi are currently studied in combination with checkpoint blockers in patients with metastatic and unresectable HER2/neu negative breast cancer (138).

#### Manipulating the metabolism of myeloid cells

Because the metabolism of TIMs is linked to their function, changing metabolic pathways has been studied as a strategy to target myeloid cells (229). MDSCs have an enhanced expression of CD36, a fatty acid translocase that enables uptake and oxidation of fatty acids, and as such an increase in their immunosuppressive capacity (230). Inhibiting fatty acid oxidation decreases the immunosuppressive capacity of MDSCs, and moreover mediates antitumor effects in experimental models when combined with low-dose CT and adoptive cell therapy (231). Metabolic pathways were also studied in TADCs. Fatty acid metabolism in DCs has been linked to their ability to cross-present antigens, a critical process to stimulate tumor-specific CD8^+^ T cells (232). Nonetheless, enhanced levels of lipids as observed in several preclinical mouse cancer models and cancer patients has a negative impact on DC cross-presentation. Normalization of lipid levels in these cancer models restored the functional activity of lipid rich DCs and enabled them to become more potent when used in a cancer vaccine (233). It was further shown that anaerobic glycolysis after TLR stimulation is essential to upregulate among others co-stimulatory molecules and IL-12 (234, 235). Also the metabolic signature of TAMs is correlated to their function (236, 237). The direct link between TAM metabolism, tumor vasculature and metastasis, was recently shown using a genetic approach. Herein genetic deletion of regulated in development and DNA damage responses 1 (REDD1), an inhibitor of mTOR, which is highly expressed by TAM2 was shown to increase glucose uptake and direct TAM2 toward glycolysis (238), a hallmark of TAM1 (239). Consequently, TAMs competed with tumor endothelial cells for glucose consumption, which resulted in stabilization of the tumor vasculature, thereby preventingmetastasis (238).

#### Manipulating distinct tumor promoting functions

Several enzymes expressed by TIMs have been inextricably connected to their immunosuppressive function, among which IDO, ARG-1, iNOS, and COX2, and have therefore been a focus of research. IDO is a tryptophan degrading enzyme that can be induced in MDSCs, TADCs, TAMs, and to some extent TANs in response to stimuli such as pro-inflammatory cytokines, TLR ligands, hormones, PGE2, and contact dependent stimuli such as B7:cytotoxic T lymphocyte antigen 4 (CTLA-4) interactions (10, 240–242). The net effect of IDO mediated conversion of tryptophan to kynurenine is induction of T cell anergy, apoptosis, and commitment of CD4^+^ T cells toward immunosuppressive Tregs (243). These Tregs can further amplify the immunosuppressive TME by endowing TADCs with tolerogenic properties (e.g., upregulation of IDO), and by recruiting and activating MDSCs, which exploit expression of IDO, ARG-1, and iNOS in addition to immunosuppressive cytokines and co-inhibitory molecules to suppress T cells (244). The tryptophan catabolism is therefore an important mechanism of cancer immune escape. Expression of IDO has been linked to poor clinical prognosis in several cancers, as it correlates with decreased survival as well as increased risk of metastasis (245). Therefore, strategies to disrupt IDO's enzymatic activity or expression have been studied extensively. In animal studies, genetic inhibition of IDO expression resulted in infiltration, and activation of granulocytes that established a TME favoring tumor growth (246). The tyrosine kinase inhibitor imatinib, which inhibits COX2 activity, therefore blocks PGE2 production a potent stimulus for IDO upregulation, has been successfully used in the context of IDO inhibition (247, 248). Also small-molecule inhibitors of IDO exhibit anticancer activity and cooperate with immunotherapy, RT or CT to trigger rapid regression of aggressive tumors otherwise resistant to treatment (249). Several IDO inhibitors are under clinical evaluation either as a mono- or combination therapy (e.g., checkpoint blockade and CT) (250). The IDO inhibitors most intensively studied in clinical trials are epacadostat and indoximod. In a phase I trial, epacadostat showed to normalize kynurenine levels in patients with advanced solid malignancies (251). In phase I-III trials, epacadostat showed promising results in combination with various checkpoint inhibitors, including durvalumab (anti-PD-L1), pembrolizumab (anti-PD-1), and ipilimumab (anti-CTLA-4), in different cancer types (252). Indoximod was shown to counteract the immunosuppressive effects of kynurenine, to activate multiple immune cells and to stimulate Treg to T_H_1 conversion (253–255). Ongoing phase II-III clinical studies investigate the effect of indoximod in combination with the checkpoint inhibitor nivolumab (anti-PD-1) in patients with metastatic melanoma. Further development resulted in a compound, NLG802, the prodrug of indoximod, which in animal studies induced antitumor responses at lower doses than indoximod. Recently, NLG802 entered phase I clinical testing (256).

ARG-1 is another amino acid degrading enzyme. It converts L-arginine into L-ornithine and urea, and can be upregulated in MDSCs, TAM2 and tolDCs by multiple factors, including PGE2 (produced by COX2), GM-CSF, TGF-β, IL-6, and IL-10, where the latter two both act via STAT3 (257–260). Also hypoxia has been linked to ARG-1 expression (261). Expression of ARG-1 by myeloid cells has been unambiguously linked to tumor promoting activities, and L-ornithine was shown to further stimulate alternative activation of myeloid cells (262–264). iNOS is also an L-arginine converting enzyme, which produces NO and citrulline as a result. Expression of iNOS has been shown in TAM1, inflammatory DCs and MDSCs in response to IL-1β, IL-6, IFN-γ, TNF-α, and TLR4 agonists, and has been linked to both anti- and protumor activities (265, 266). This dual role of iNOS and NO is likely depending on the levels, source and timing of NO production, and is further influenced by the TME. In early events of tumor development some myeloid cells like macrophages can generate high concentrations of NO, thereby inducing tumor cell apoptosis. However, in an established TME, myeloid cells are reprogrammed to support tumor progression. At that time low amounts of NO act pro-angiogenic and enhance tumor growth and metastasis by inducing growth factors such as VEGF and MMPs. Moreover, a temporal relationship between the expression of ARG-1 and iNOS in TIMs such as macrophages has been linked to immunosuppression. It was proposed that ARG-1 occurs prior to iNOS expression to stop T cell proliferation and that subsequent iNOS expression and as such NO release, stimulates T cell apoptosis, thereby ending the anticancer immune responses (267). Therefore, strategies to block ARG-1 and iNOS have been developed. Inhibitors like nor-N-hydroxy-L-arginine and aminoguanidin (or 1400W) downregulate ARG-1 and iNOS activity in MDSCs, restore T cell antitumor immunity and reduce tumor progression (176, 268). Moreover, recent studies showed that combining ARG-1 inhibition with immune checkpoint therapy or RT is a promising strategy (244, 269). Several reports recommend targeting both ARG-1 and iNOS to augment the therapeutic effect (270). Phosphodiesterase-5 (PDE5) inhibitors, like sildenafil, tadalafil, and vardenafil, were shown to decrease ARG-1 and iNOS expression. Consequently the suppressive activity of MDSCs was blocked which ultimately boosted tumor-specific T cell responses (271–273). Similarly, NO-releasing aspirin was shown to reduce ARG-1 and iNOS, and enhance the number and function of tumor-specific T cells (274). The prior use of PDE5 inhibitors and NO-releasing aspirin in other indications prompted their use in clinical trials, often in combination with other cancer therapies including RT, CT, and immunotherapy (vaccination, immunomodulatory drugs and immune checkpoint blockade) (275). While many trials are ongoing, pilot trials in metastatic melanoma patients report on its safety and further show a decreased frequency and function of MDSCs, correlating with an increased T cell reactivity and improved clinical outcome (276–278). While these studies mainly focused on the role of MDSCs, it is conceivable that these agents could also modulate other ARG-1 and iNOS expressing myeloid cells like TAMs and TADCs.

COX2 is the enzyme responsible for the metabolic conversion of arachidonic acid into various prostaglandins including PGE2, which has an immunosuppressive function as evidenced by its ability to induce IDO and ARG-1 expression in various TIMs. Another link to immunosuppressive amino acid degrading enzymes and COX2 in myeloid cells is the observation that NO is involved in regulating COX2 expression (279). Inhibition of COX2 in mouse and human myeloid cell progenitors was shown to inhibit MDSC and TAM2 generation, and favor differentiation toward mature APCs (280–283). Genetic deletion of COX2 in myeloid cells *in vivo* was shown to reduce macrophage infiltration, increase T cell numbers in the tumor and linked herewith reduce tumor progression (284). Pharmacological inhibition of COX2 was also shown to delay tumor growth in various studies and even synergize with immunotherapy. In these studies, COX2 inhibition was linked to reduced MDSC accumulation, and reduced IDO or ARG-1 expression (248, 285–288).

Overall, inhibition of key immunosuppressive enzymes was shown to (partially) revert the immunosuppressive TME and as such facilitate cancer specific immunity. Future preclinical and clinical studies will have to substantiate if targeting one or more of the suppressive mechanisms exerted by TIMs suffices to control cancer progression.

## Conclusion

In this review we emphasize the multifaceted functions that TIMs possess to support cancer manifestation, progression, and metastasis next to the roadblocks they form to successful therapeutic interventions. This is evidenced by the abundance of publications on the subject of TAMs, TADCs, TANs, MDSCs, and the commonalities they share such as plasticity and immunosuppressive functions. So far, vast efforts has been invested in the development of pharmacological compounds targeting TIMs to alter their cell number or repolarize their functions. These strategies resulted in significant therapeutic benefits in several preclinical models and are currently clinically evaluated as additional modalities in cancer treatment.

Nonetheless, caution is warranted when neutralizing immune factors that are not unique to the tumor and its TME (e.g., PGE2, ROS, iNOS, or TGF-β), since they are indispensable in maintaining homeostasis. Therefore, TIM depleting or function altering therapies targeting one of these factors, must be transient or local in nature. For future therapies, it will be essential to expand our knowledge on mechanisms that drive the immunosuppressive barricades herded by TIMs. Also, the type of cancer should be considered since some myeloid compositions can be beneficial in one type while unfavorable in another. Since migration patterns and metabolic aberrations occur during the several stages of tumor progression, it is important to push current methodology toward a combination of functional imaging and advanced in silico analysis. This will be essential to understand and predict the dynamics of the TME as well as why some predicted therapies underperform *in vivo*.

Conventional therapies are often focused on eliminating a particular subset among the abundance of myeloid cells neglecting those that are desirable. Tumor lyses, clearance and antigen presentation are essential modalities that are especially designated to myeloid cells, however they are still undervalued. Instead of eradicating a complete TIM population we rather suggest to mold the myeloid cell composition and polarization state, by combining several myeloidal immunotherapies, based on the redundancy and commonalities in TIM phenotype and function. In conclusion, we believe that reorganizing the myeloid landscape can clear away the roadblock to a successful cancer therapy.

## Author contributions

All authors listed have made a substantial, direct and intellectual contribution to the work, and approved it for publication.

### Conflict of interest statement

The authors declare that the research was conducted in the absence of any commercial or financial relationships that could be construed as a potential conflict of interest.
